# Peroxisome proliferator-activated receptor γ ligand-induced growth inhibition of human hepatocellular carcinoma

**DOI:** 10.1054/bjoc.2001.1821

**Published:** 2001-06

**Authors:** M A K Rumi, H Sato, S Ishihara, K Kawashima, S Hamamoto, H Kazumori, T Okuyama, R Fukuda, N Nagasue, Y Kinoshita

**Affiliations:** Second Department of Internal Medicine, Shimane Medical University; Second Department of Surgery, Shimane Medical University, Shimane 693-8501, Izumo, Japan

**Keywords:** PPARγ, hepatocellular carcinoma, growth inhibition, cell cycle arrest

## Abstract

Peroxisome proliferator-activated receptor γ (PPARγ) ligands have been implicated in the growth inhibition and differentiation of certain human cancers with diverse tissue origin. In this study, expression of PPARγ in human hepatocellular carcinoma (HCC) and the effect of PPARγ ligands on HCC cells were investigated in vitro using Hep G2, HuH-7, KYN-1 and KYN-2 cell lines. All cell lines were found to express functionally active PPARγ and a marked growth inhibition was induced by thiazolidinedione ligands troglitazone, and pioglitazone as well as with its natural ligand 15-deoxy-Δ^12,14^-prostaglandin J _2_. The growth inhibitory effect was associated with a dose-dependent inhibition of DNA synthesis, cell cycle progression and α fetoprotein expression. © 2001 Cancer Research Campaign http://www.bjcancer.com


*British Journal of Cancer* (2001) **84**(12), 1640–1647
© 2001 Cancer Research Campaign
doi: 10.1054/ bjoc.2001.1821, available online at http://www.idealibrary.com on IDEAL®

### Peroxisome proliferator-activated receptor γ ligand-induced growth inhibition of human hepatocellular carcinoma

**MAK Rumi[1], H Sato[1], S Ishihara[1], K Kawashima[1], S Hamamoto[1], H Kazumori[1], T Okuyama[1], R Fukuda[1], N Nagasue[2] and Y Kinoshita[1]**

[1]Second Department of Internal Medicine, Shimane Medical University; [2]Second Department of Surgery, Shimane Medical University, Izumo, Shimane 693-8501, Japan

**Summary** Peroxisome proliferator-activated receptor γ (PPARγ) ligands have been implicated in the growth inhibition and differentiation of certain human cancers with diverse tissue origin. In this study, expression of PPARγ in human hepatocellular carcinoma (HCC) and the effect of PPARγ ligands on HCC cells were investigated in vitro using Hep G2, HuH-7, KYN-1 and KYN-2 cell lines. All cell lines were found to express functionally active PPARγ and a marked growth inhibition was induced by thiazolidinedione ligands troglitazone, and pioglitazone as well as with its natural ligand 15-deoxy-$\Delta^{12,14}$-prostaglandin $J_2$. The growth inhibitory effect was associated with a dose-dependent inhibition of DNA synthesis, cell cycle progression and α fetoprotein expression. © 2001 Cancer Research Campaign http://www.bjcancer.com

**Keywords**: PPARγ; hepatocellular carcinoma; growth inhibition; cell cycle arrest

Peroxisome proliferator-activated receptor γ (PPARγ), a member of the nuclear hormone receptors, can act as ligand-sensitive transcription factor (Mangelsdorf et al, 1995; Kliewer and Willson, 1998). Activated receptors heterodimerize with retinoid X receptor (RXR) and can alter transcription of target genes after binding to peroxisome proliferator responsive elements (PPRE) (Kliewer et al, 1992). PPARγ was initially reported for its regulatory roles in insulin sensitization and adipocyte differentiation (Chawla et al, 1994; Tontonoz et al, 1994a, 1994b). However, later studies have shown that PPARγ is also expressed in other cell types and it has recently been of interest for its role in cell proliferation and cancer.

In vitro studies have revealed the growth inhibitory effects of PPARγ ligands on human cancer cells of different tissue origin, including liposarcoma (Tontonoz et al, 1997), adenocarcinoma of breast (Elstner et al, 1998; Mueller et al, 1998; Clay et al, 1999), colorectal adenocarcinoma and carcinoma (Sarraf et al, 1998; Brockman et al, 1998; Kitamura et al, 1999), gastric adenocarcinoma (Takahashi et al, 1999; Sato et al, 2000), pancreatic carcinoma (Motomura et al, 2000), adenocarcinoma and carcinoma of prostate (Kubota et al, 1998; Butler et al, 2000), transitional epithelial cancer of urinary bladder (Guan et al, 1999), chorio carcinoma of placenta (Keelan et al, 1999), carcinoma of lung and non-small cell lung cancer (Chang and Szabo, 2000; Tsubouchi et al, 2000) and myeloid leukaemias (Asou et al, 1999; Hirase et al, 1999; Sugimura et al, 1999). In vivo study on xenograft of human tumours in immunodeficient mice followed by troglitazone treatment also provided similar results (Elstner et al, 1998; Kubota et al, 1998; Sarraf et al, 1998). Ligand-mediated PPARγ activation in those cancer cells induced cell cycle arrest (Tontonoz et al, 1997; Brockman et al, 1998; Sugimura et al, 1999; Motomura et al, 2000), differentiation (Tontonoz et al, 1997; Kubota et al, 1998; Mueller et al, 1998; Sarraf et al, 1998; Chang and Szabo, 2000), and apoptosis (Elstner et al, 1998; Clay et al, 1999; Keelan et al, 1999; Sato et al, 2000) or nonapoptotic cell death (Kubota et al, 1998; Butler et al, 2000). Recently, troglitazone has been used in clinical trial for the patients with advanced liposarcoma (Demetri et al, 1999) and for patients with advanced prostate cancer (Hisatake et al, 2000; Mueller et al, 2000). The drug was found to induce histologic and biochemical differentiation in liposarcoma and a prolonged stabilization of prostate-specific antigen (PSA) level in prostate cancer patients. Such results suggest that ligands of PPARγ may serve as a biological modifier in human cancers and the therapeutic potential should be further investigated. Human liver tissue has been reported to express PPARγ (Auboeuf et al, 1997; Vidal-Puig et al, 1997), however, expression of PPARγ in human hepatocellular carcinoma (HCC) and the effect of PPARγ agonists have not yet been studied and this study was designed to investigate that.

PPARγ can be activated by certain polyunsaturated fatty acids (Kliewer et al, 1997; Xu et al, 1999), prostaglandin $J_2$ metabolite 15-deoxy-$\Delta^{12,14}$-$PGJ_2$ (15d-$PGJ_2$) (Forman et al, 1995; Kliewer et al, 1995), the thiazolidinedione (TZD) class of antidiabetic drugs (Lehmann et al, 1995; Elbrecht et al, 1996) and a variety of nonsteroidal anti-inflammatory drugs (Lehmann et al, 1997). Recent attention has focused on troglitazone because of its rare but potentially lethal hepatotoxicity (Watkins and Whitcomb, 1998; Kohlroser et al, 2000) although at present, no similar side effects have been observed with other TZD, either rosiglitazone or pioglitazone (Henney, 1999). Because evidence to date does not indicate that hepatotoxicity is attributable to TZDs as a class or to PPARγ agonists in general, in vitro growth inhibitory effects of PPARγ

*Received 7 July 2000*
*Revised 27 February 2001*
*Accepted 28 February 2001*

*Correspondence to:* H Sato ligands were studied on human HCC cells in view of evaluating their potential therapeutic application.

### MATERIALS AND METHODS

### Cell lines and culture condition

Hepatocellular carcinoma cell lines Hep G2 was obtained from American Type Culture Collection (Manassas, VA, USA), HuH-7 from JCRB Cell Bank (National Institute of Health Sciences, Osaka, Japan) whereas, KYN-1 and KYN-2 were kind gifts from Prof M Kojiro (Department of Pathology, Kurume University School of Medicine, Japan). Hep G2 (Aden et al, 1979), as well as HuH-7 (Nakabayashi et al, 1982) was established from well differentiated hepatocellular carcinoma, KYN-1 (Yano et al, 1986) from a moderately differentiated, and KYN-2 from a pleomorphic hepatocellular carcinoma corresponding to Edmondson-Steiner grade III (Yano et al, 1988). All the hepatocellular carcinoma cell lines secrete albumin and AFP, in addition, KYN-1 possesses the nature of transformation to adenocarcinoma with production of mucicarmin-positive materials (Yano et al, 1986). Cells were grown at 37˚C in Dulbecco's Modified Eagle Medium (DMEM) (Nissui Pharmaceutical, Tokyo, Japan) supplemented with 10% fetal bovine serum (ICN Biomedicals, Aurora, Ohio, USA), L-glutamine (ICN Biomedicals), penicillin-streptomycin (ICN Biomedicals) and maintained in an incubator with 5% $CO_2$ and constant humidity.

Sensitivity to PPARγ ligands was studied in all the cell lines. However, changes in cell cycle distribution caused by PPARγ ligands, as well as protein level of cyclin-dependent kinase (CDK) inhibitors and α fetoprotein (AFP) or albumin mRNA expression after troglitazone treatment were investigated using the representative cell line Hep G2.

### Experimental drugs

Troglitazone (±)-5-[4-(6-hydroxy-2,5,7,8-tetramethyl-chroman-2-ylmethoxy) benzyl]-2,4-thiazolidinedione, MW 441.55, was kindly provided by Sankyo Pharmaceuticals Co (Tokyo, Japan) and Pioglitazone (±)-5-[4-[2-(5-ethyl-2-pyridil) ethoxy]benzyl]-thiazolidine-2,4-dione monohydrochloride (AD-4833 HCl), MW 392.90, by Takeda Chemical Industries, Ltd (Tokyo, Japan). 15d-$PGJ_2$, 15-deoxy-delta 12,14-prostaglandin J2, MW 316.4, was purchased from Calbiochem (Calbiochem-Novabiochem Corporation, La Jolla, CA, USA). All the experimental drugs were prepared fresh before use, dissolving troglitazone or pioglitazone in DMSO and 15d-$PGJ_2$ in ethanol according to manufacturers instruction. After dissolving the drugs in respective vehicles and serially diluted, they were mixed into complete media to obtain the desired concentration of the experimental drugs in solution and then applied to the growing adherent cells. Vehicle concentration in the medium was maintained 0.1% v/v for all.

A dose ranging from 0.1 to 50 μM troglitazone was selected for this in vitro study according to the pharmacokinetics (Plosker and Faulds, 1999) and tissue distribution of the drug (Kawai et al, 1997). For comparison of the dose effect, the 2 other PPARγ ligands, pioglitazone and 15d-PGJ2 were also applied to cells at the same dosages.

### Assays for PPARγ expression

#### *RT-PCR*

PPARγ expression at mRNA level was investigated by RT-PCR. From each cell line, total RNA was extracted by Isogen (Nippon Gene, Tokyo, Japan). 7.5 μg of total RNA was reverse transcribed with oligo dT primer in a 50 μl reaction using ProstarTM First-Strand RT-PCR kit (Stratagene Cloning Systems, La Jolla, CA, USA). 1 μl of the cDNA was amplified by PCR on GeneAmp PCR system 9600 (PE Applied Biosystems, Foster City, CA, USA). Amplification was carried out in a 25 μl reaction volume using 10 pmol of each of the primers (sense-5′TCTGGCCCAC-CAACTTTGGG 3′ and antisense-5′ CTTCACAAGCATGAACT-CCA 3′) (Kubota et al, 1998) with 200 μM each dNTP, 1.5 mM $MgCl_2$ and 0.75 unit of Amplitaq gold (PE Applied Biosystems) for 30 cycles (94˚C 30 s, 56˚C 30 s and 72˚C 60 s). 5 μl of PCR products were electrophoresced on 1.5% agarose gel with 0.5% ethidium bromide and visualized on UV. As internal control, β-actin cDNA sequence was amplified using the human β-actin control amplimer set 5′ ATCTGGCACCACACCTTCTACAAT-GAGCTGCG 3′ (sense) and 5′ CGTCATACTCCTGCTTGCT-GATCCACATCTGC 3′ (antisense) (Clontech Laboratories, Palo Alto, CA, USA). In all cases RNA processed without reverse transcriptase and preparation without template was used to check carryover and amplified products purified by Geneclean (Bio 101, Carlstad, CA, USA) were confirmed by direct sequencing on ABI prism 310 Genetic Analyser (PE Applied Biosystems) using BigDye Terminator Cycle Sequencing kit (PE Applied Biosystems).

#### *Western blot*

Cells were lysed in 20 mM Tris, pH 7.6 containing 0.1% SDS, 1% Triton-X 100, 1% deoxycholate and 100 μg $ml^{-1}$ protease inhibitor PMSF and proteins were extracted as described (Maekawa et al, 1997). Protein concentration was estimated in Bradford method using BioRad Protein Assay Reagent (Bio-Rad, Hercules, CA, USA). From each cell line, 50 μg of protein was separated by 12.5% SDS-PAGE (Multigel, Daiichi Pure Chemicals, Tokyo, Japan) and transferred to polyvinylidene diflouride (PVDF) membrane (Hybond-P, Amersham Pharmacia Biotech, Buckinghamshire, UK). After 2 h blocking the membrane with 5% skim milk (Difco Laboratories, Detroit, MI, USA) in TBS (20 mM Tris, 150 mM NaCl, pH 7.6) and overnight incubation with mouse anti-human PPARγ monoclonal antibody (sc-7273, Santa Cruz Biotechnology, Santa Cruz, CA, USA) in 1:500 dilution at 4˚C, it was reacted with peroxidase-conjugated anti-mouse IgG (1:2000) (Dako Corporation, Carpinteria, CA, USA) washed in TTBS (TBS plus 0.05% Tween 20) followed by TBS and resulting signals were imaged by enhanced chemiluminescence (ECL) detection system (Amersham Pharmacia Biotech).

### PPARγ functional assay

To investigate whether PPARγ expressed in HCC cell lines are functionally active, we measured the reporter gene activity using luciferase assay (Alam and Cook, 1990; Brasier et al, 1989; Nordeen, 1988). Induction of the transcriptional activation by PPARγ ligand troglitazone through PPRE (Kliewer et al, 1992; Juge-Aubry et al, 1997) was assessed by the luciferase assay after transfecting Hep G2 cells a eukaryotic expression vector of firefly luciferase with PPRE cloned upstream to its SV40 promoter. PPRE sequence from the acyl-CoA oxidase promoter was

© 2001 Cancer Research Campaign 

synthesized as described (He et al, 1999). Oligonucleotides 5′ GATCCGGACCAGGACAAAGGTCACGTTCGGACCAGGA-CAAAGGTCACGTTCGTCCTQQTCCQ3′ and 5′GATCCGAA-CGTGACCTTTGTCCTGGTCCGAACGTGACCTTTGTCCTG GTCCG 3′ were annealed and 2 copies were cloned into the multiple cloning site (MCS) upstream to SV40 promoter of the PicaGene promoter vector (PGV-P2) (Nippon Gene) to construct PGVP2.PPRE. Standard dual luciferase assay was performed in 24-well plates, transfecting $10^5$ cells $well^{-1}$ with 1 µg of PGVP2.PPRE and 0.05 µg of PicaGene SeaPansy TK control vector (pRL-TK) (Nippon Gene) using DOTAP liposomal transfection reagent (Roche Molecular Biochemicals, Mannheim, Germany). 10 h after the transfection, fresh media containing 10 µM troglitazone or vehicle only was added to the transfected cells. After 12 h treatment with troglitazone, cells were lysed with passive lysis buffer and luciferase assay was performed with PicaGene Dual SeaPansy Luminescence Kit (Nippon Gene) on Lumat LB9506 luminometer (EG & G Berthhold, Bad Wildbad, Germany). Results were evaluated after normalization of the firefly luciferase activity of PGVP2.PPRE with that of sea pansy luciferase activity of pRL-TK. Tests were performed in duplicate, repeated twice and the mean value of increment in luciferase activity were analysed.

### Assessment of growth inhibition

### *Viable cell counting*

Effect on cell growth was evaluated by direct cell counting using a haemocytometer. $5 \times 10^5$ cells were seeded into 60 mm plates and after 24 h, PPARγ ligand troglitazone, pioglitazone or 15d-$PGJ_2$ was added to culture media in 10, 25 and 50 µM concentration. After 48 h, both floating and adherent cells were harvested and viable cells were counted by trypan blue dye exclusion. Relative rate of increment in cell counts in presence of ligands compared with that of control without drug was considered representative of cell growth.

### *Assay for DNA synthesis*

DNA synthesis was assessed by $^3$H-thymidine incorporation. $10^5$ cells were seeded into 24-well plates and after 24 h culture in complete media, experimental drugs in varying concentrations dissolved in media was added to the growing cells. After 24 h treatment with drug, 1 µCi [methyl-$^3$H]-thymidine (Amersham Pharmacia Biotech) was added to each well and incubated for a further 6 h. Then the cells were trypsinated and harvested onto a glassfibre filtermat by a cell harvester, dried 1 h and $^3$H-thymidine incorporation was measured with 1450 Microbeta™ scintillation counter (Wallac Oy, Turku, Finland). Assays were performed in triplicate, the mean CPM values after normalization were analysed for relative $^3$H-thymidine incorporation and repeated thrice.

### Cell cycle analysis

Cell-cycle analysis was performed on the representative Hep G2 cells. Approximately $2 \times 10^6$ cells were seeded in 100 mm plates and on growing cells, fresh media with experimental drugs in various concentrations was added. Depending on the results of viable cell counts and $^3$H-thymidine incorporation assay results, troglitazone and pioglitazone was used in 10, 25 and 50 µM and 15d-$PGJ_2$ in 5, 10 and 25 µM. After 48 h drug treatment, cells were harvested, washed with PBS and fixed overnight in cold (– 20˚C) 75% ethanol. Ethanol fixed cells were washed twice with ice cold PBS, and then approximately equal number of control or drug treated cells were treated with 200 µg $ml^{-1}$ RNase for 60 min at 37˚C and stained with 100 µg $ml^{-1}$ propidium iodide 30 min at room temperature in dark. Cellular DNA contents were analysed by flow cytometry (EPICS Elite, Coulter Electronics, FL, USA). In each case, histogram of DNA contents in 10 000 cells were analysed using Multicycle AV software (Phoenix, San Diego, CA, USA) to evaluate relative distribution of cells in G1, S and G2 phase. Cell cycle analysis on Hep G2 cells was performed in duplicate and repeated 3 times for each drug.

### Expression of cyclin-dependent kinase (CDK) inhibitors

The effect of troglitazone on the expression of CDK inhibitor $p27^{Kipl}$, $p21^{Cipl/Wafl}$ and $p18^{Ink4c}$ was studied in Hep G2 cells by Western blot analysis. Hep G2 cells were treated with 5, 10, 25 and 50 µM troglitazone or vehicle only and total proteins were extracted 48 h after drug treatment. 50 µg of protein were separated by 15–25% gradient SDS-PAGE (Multigel, Daiichi Pure Chemicals) and transferred to PVDF membrane (Hybond-P, Amersham Pharmacia Biotech). Then the membranes were blocked with 5% skim milk in TBS at room temperature for 2 h, and reacted overnight at 4˚C on a rotatory shaker with primary mouse monoclonal antibody to human $p27^{Kipl}$ (1:500) (sc-1641, Santa Cruz Biotechnology), or $p21^{Cipl/Wafl}$ (1:500) (OP64, Oncogene Research Products, Calbiochem-Novabiochem, International) or $p18^{Ink4c}$ (1:100) (sc-9965, Santa Cruz Biotechnology) or β-actin (1:5000) (AC-15, Sigma Chemical Co, St Louis, MO, USA). After washing the membranes in TTBSmilk, reacted with HRP-conjugated anti-mouse IgG (1:1500 for anti-CDK inhibitors or 1:3000 for anti-β-actin) (Dako Corporation) for 2 h at room temperature, washed in TTBS followed by TBS and ECL detection reagent (Amersham Pharmacia Biotech) was used to visualize the signals from immune complexes.

### Northern blot analysis for albumin and AFP expression

Whether troglitazone induced differentiation in hepatocellular carcinoma cells, was investigated after 2 × 48 h pulse exposure of Hep G2 cells with troglitazone in varying concentrations. Extracted total RNA was used in Northern blot analysis for the quantitative comparison in the expression of albumin and AFP. Briefly, 10 µg of total RNA was electrophoresced in formaldehyde-containing 1% agarose gel and transferred to nitrocellulose membrane (Hybond-C Extra, Amersham Pharmacia Biotech), followed by UV cross-linking (Funa-UV-linker, Funakoshi, Tokyo, Japan). $^{32}$P-labelled (Multiprime DNA labelling systems, Amersham Pharmacia Biotech) probes used for the Northern blot analysis were a 422 bp cDNA fragment of human albumin (Niwa et al, 1996), a 395 bp cDNA fragment of human AFP (Niwa et al, 1996) and the 838 bp cDNA fragment of human β-actin (RT-PCR, internal control). After 4 h prehybridization, hybridization was carried out for 20 h at 42˚C and then membranes were washed twice at 37˚C in 2 × SSC containing 0.2% SDS for 10 min and once at 50˚C in 0.1 × SSC containing 0.2% SDS for 30 min. Autoradiography was performed using FUJIX BAS-2000 II (Fuji Photo Film, Tokyo, Japan) and photographed with a FUJIX Pictrography 3000 (Fuji Photo Film). The relative intensities of message bands were quantified using the FUJIX-BAS 2000 II Image Analyser.

© 2001 Cancer Research Campaign

### Statistical analysis

Data were expressed as mean ± SE. Values were compared and significant differences between means were determined by Analysis of Variance (ANOVA). Multiple comparisons were done by Least Significant Difference (LSD) test after ANOVA. *P* values < 0.05 were considered significant.

### RESULTS

### HCC cell lines expressed PPARγ

RT-PCR analysis readily detected the expression of PPARγ mRNA in HCC cell line Hep G2, HuH-7, KYN-1 and KYN-2 (Figure 1A). Amplified fragment was appropriate for 360 bp (Kubota et al, 1998) and direct sequencing also confirmed that RT-PCR products were identical to human PPARγ cDNA sequence (Elbrecht et al, 1996). Western blot analysis with anti-human PPARγ monoclonal antibody also detected expression of PPARγ in all HCC cell lines (Figure 1B) with molecular mass of approximately 54 kDa (Elbrecht et al, 1996).

### Expressed PPARγ was functional

Transactivation experiment showed that 10 μM troglitazone induced about 2.2 to 3.8 fold enhanced luciferase activity (Figure 1C) in all the HCC cell lines transfected with a luciferase reporter vector containing PPRE upstream to SV40 promoter. Although induction of luciferase activity was relatively lower in KYN-1 and KYN-2, the differences were not significant.

### PPARγ ligands induced growth inhibition

Trypan blue staining and direct counting of viable cells 48 h after troglitazone treatment at 10, 25 and 50 μM concentration showed a significant decrease in relative increment cell number in all cell lines (Figure 2A). With equivalent dose, 15d-$PGJ_2$ also reduced the viable cell count of Hep G2 significantly (Figure 2B). However, at higher concentration (i.e. 25 and 50 μM) of 15d-$PGJ_2$ and troglitazone, cell death was marked. Pioglitazone induced inhibition of cell growth was quantitatively less than that of troglitazone and 15d-$PGJ_2$ as observed on Hep G2 cells (Figure 2B).

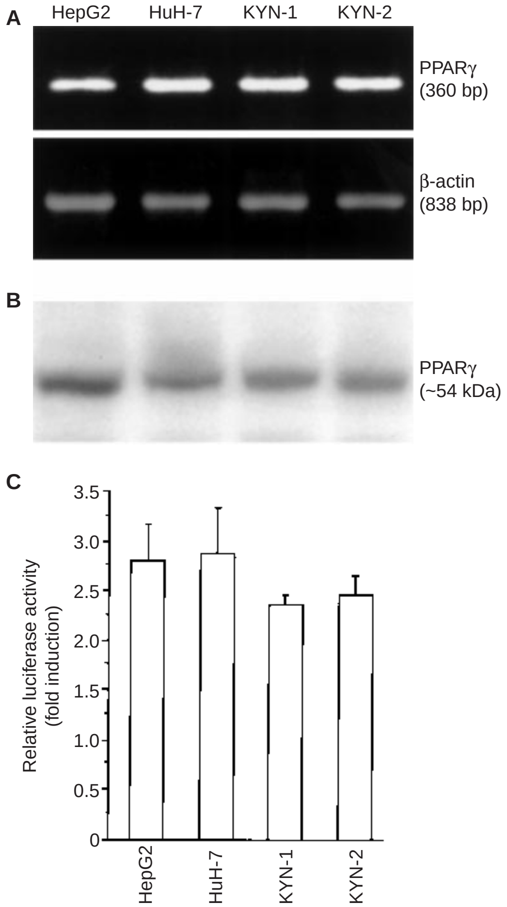

**Figure 1** PPARγ is expressed and functionally active in human hepatocellular carcinoma cell lines. (**A**) PPARγ expression at mRNA level. Total RNA extracted from the cell lines was reverse transcribed and cDNA was subjected to PCR for PPARγ amplification. β-actin cDNA was also amplified as internal control. (**B**) PPARγ protein expression in hepatocellular carcinoma cell lines detected by Western blot using monoclonal anti-hPPARγ. (**C**) PPRE transactivation in hepatocellular carcinoma cells: reporter gene assay showed that a 12 h activation with 10 μM troglitazone induced a significantly enhanced expression (2.2–3.8 fold induction) of luciferase in all cell lines

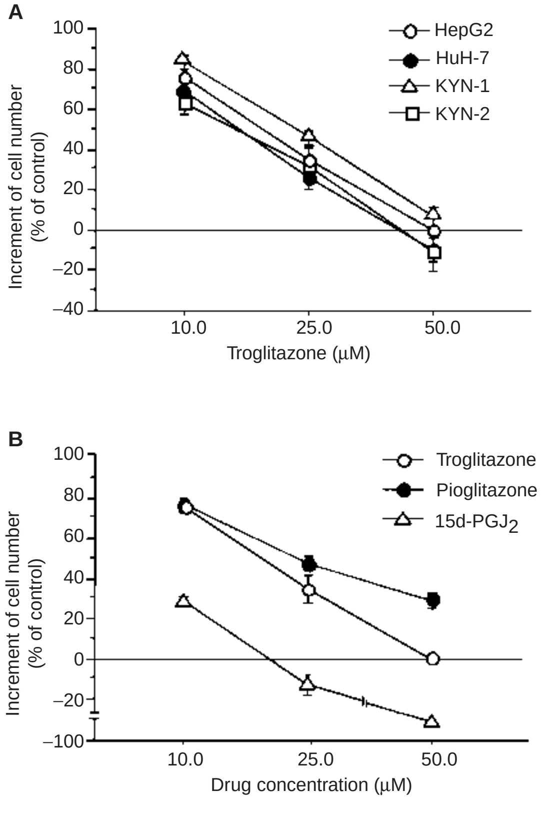

**Figure 2** Effect of PPARγ ligands on proliferation of hepatocellular carcinoma cell lines. Cells were incubated in presence or absence (control) of PPARγ ligands and after 48 h, total number of viable cells was counted by trypan blue dye exclusion. Increment of cell number with drugs is expressed compared with that of control. Each value indicates the mean ± SE of 3 experiments in duplicate. (**A**) Troglitazone dose dependently induced a significant inhibition in increment of cell number in all 4-cell lines ($P < 0.006$). (**B**) Effects of different PPARγ ligands on HepG2 cells, showing that pioglitazone and 15d-$PGJ_2$ also significantly inhibited the cell number increment ($P = 0.000$)

© 2001 Cancer Research Campaign

### PPARγ ligands inhibited DNA synthesis

Troglitazone induced inhibition of DNA synthesis as evidenced by $^{3}$H-thymidine incorporation in all the HCC cell lines after 30 h drug treatment. There was a significant dose-dependent decrease in $^{3}$H-thymidine incorporation with all concentrations of troglitazone used starting from 1 μM in Hep G2, HuH-7 and KYN-1 but 5 μM in KYN-2 (Figure 3A). More than 95% inhibition was observed in all cell lines at 50 μM troglitazone, about 80% at 25 μM and nearly 50% at 10 μM (Figure 3A). Pioglitazone induced inhibition of $^{3}$H-thymidine incorporation was found significant from 1 μM onward in Hep G2, KYN-1 and KYN-2 but 5 μM in HuH-7 (Figure 3B). However, 15d-PGJ$_2$ mediated significant inhibition at 1 μM in KYN-2 cells and from 5 μM in other cell lines (Figure 3C).

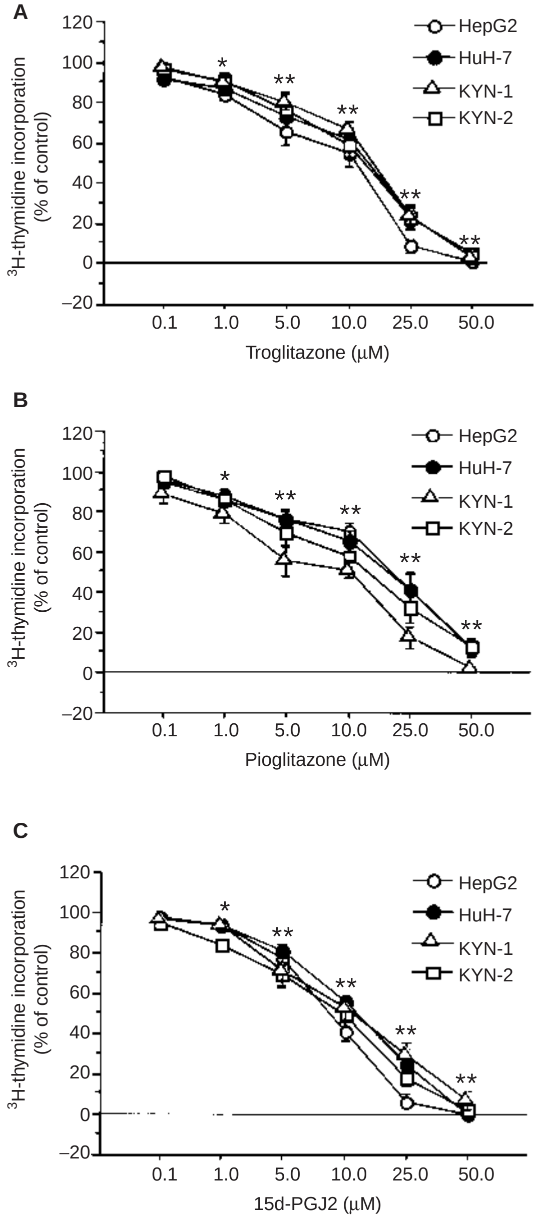

**Figure 3** PPARγ ligands induced inhibition of DNA synthesis in hepatocellular carcinoma cell lines. Cells were treated with ligands in 0.1 to 50 μM concentration and after 24 h, a standard $^{3}$H-thymidine incorporation assay was performed. Data represent mean ± SE values from 3 assays in triplicate wells. (**A**) Troglitazone dose dependently induced inhibition of $^{3}$H-thymidine incorporation in all of 4 HCC cell lines. * indicates significant inhibition ($P < 0.05$) for Hep G2, HuH-7 and KYN-1, ** for all cell lines. (**B**) Pioglitazone-induced inhibition of $^{3}$H-thymidine incorporation, * indicates significant inhibition ($P < 0.05$) for Hep G2, KYN-1 and KYN-2, ** for all cell lines. (**C**) 15d-PGJ$_2$ treatment also resulted in a similar inhibition in $^{3}$H-thymidine incorporation. * indicates significant inhibition ($P < 0.05$) for KYN-2, ** for all cell lines

### PPARγ ligands altered cell cycle progression

The cell cycle distribution changes were evident after exposure of Hep G2 cells to troglitazone, pioglitazone and 15d-PGJ$_2$ (Figure 4). With increasing dose of the ligands, cell cycle distribution significantly increased in G1 phase and decreased in S phase (Figure 4). In addition, 15d-PGJ$_2$ also induced a significant increase in G2 phase (Figure 4C). Although G2 phase increased slightly with troglitazone (Figure 4A), the dose-dependent G2 changes with troglitazone or pioglitazone were found to be statistically insignificant.

### Troglitazone increased p27$^{Kipl}$ and p18$^{Ink4c}$ protein levels

Quantitative comparison of dose-dependent changes in the CDK inhibitor levels in Hep G2 cells after troglitazone treatment is shown in Figure 5 (A, B and C). Cell cycle arrest evident at 48 h troglitazone treatment was associated with a dose-dependent increase in CDK inhibitor p27$^{Kipl}$ and p18$^{Ink4c}$, with a decline in p21$^{Cipl/Wafl}$ level.

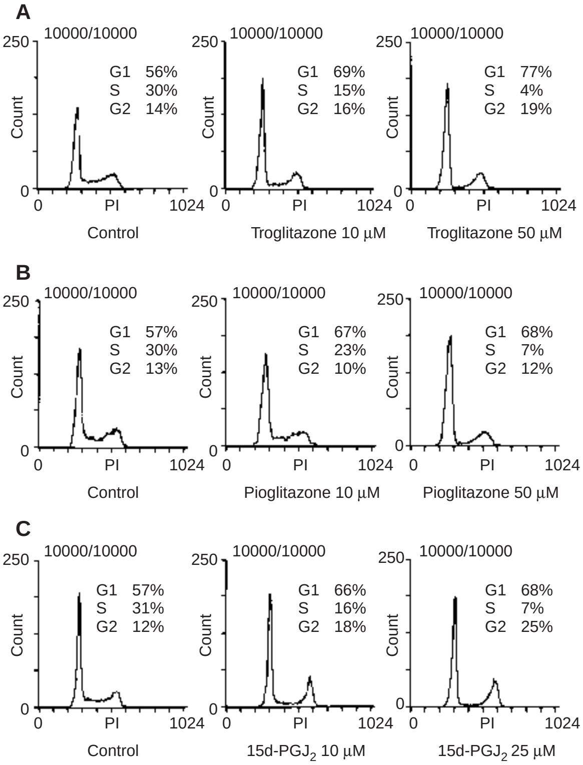

**Figure 4** PPARγ ligands induced inhibition of cell cycle progression. Histogram of propidium iodide (PI) stained DNA contents of HepG2 cells, showing a markedly increased G1 phase and decreased S phase after 48 h treatment with 0, 10 and 50 μM troglitazone (**A**), 0, 10 and 50 μM pioglitazone (**B**), 0, 10 and 25 μM 15d-PGJ$_2$ (**C**). G2 increase was also observed with troglitazone and 15d-PGJ$_2$

 *© 2001 Cancer Research Campaign*

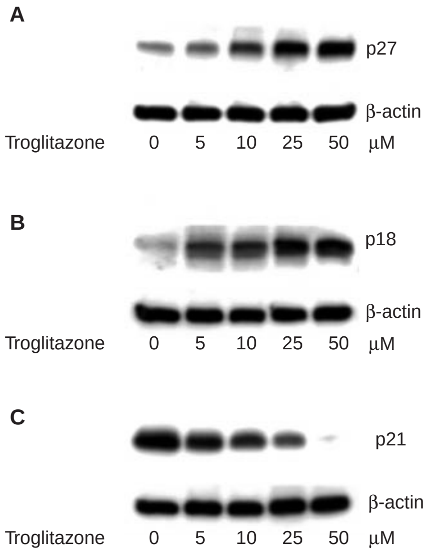

**Figure 5** Effect of troglitazone on CDK inhibitor $p27^{Kip1}$, $p21^{Cip1/waf1}$ and $p18^{Ink4c}$ protein level in Hep G2 cells. Troglitazone treatment dose dependently increased the $p27^{Kip1}$ (**A**) as well as $p18^{Ink4c}$ (**B**), with a decreased $p21^{Cip1/Waf1}$ level (**C**). 48 h after troglitazone treatment at 0, 5, 10, 25 and 50 µM concentration, proteins were extracted and Western blots were performed with mouse monoclonal antibody to human $p27^{Kip1}$, $p21^{Cip1/Waf1}$ and $p18^{Ink4c}$. As internal control, β-actin (42 kDa) was detected using a mouse monoclonal antibody. Below each lane, dosages of troglitazone are indicated

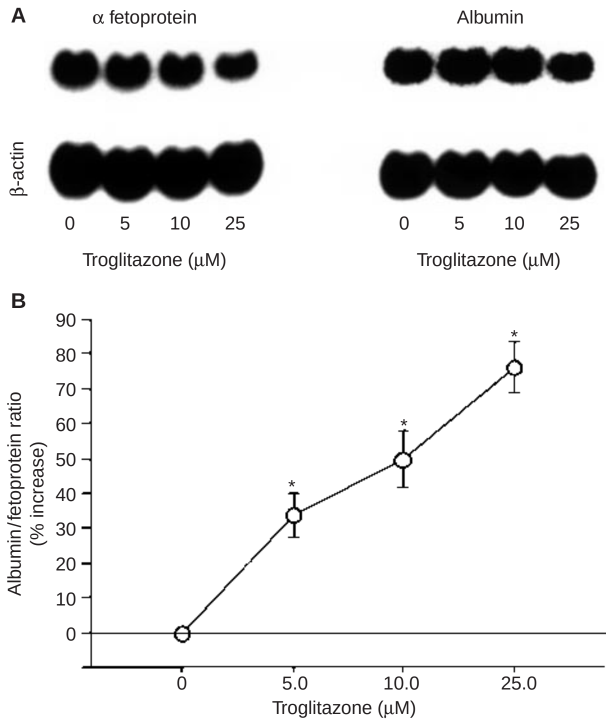

**Figure 6** Troglitazone effects on AFP and albumin expression in Hep G2 cells. (**A**) Autoradiogram of a representative Northern blot showing effects of troglitazone on AFP and albumin expression. Detection of β-actin expression was used as internal control. (**B**) Northern analysis data representing ratio of albumin and AFP message signals. Values are expressed compared to that of the control. Mean ± SE values from 4 different experiments show that troglitazone dose dependently increase the ratio of albumin/AFP expression. * indicates significant ($P < 0.05$) increase

### Troglitazone decreased AFP expression

Northern blot analysis on RNA extracted from Hep G2 cells after exposure to troglitazone, revealed that expression of AFP decreased while there was a little increase in the expression of albumin in 5 and 10 µM but decreased at 25 µM (Figure 6A). On repeated experiments, it was found that even with a decreased albumin expression at 25 µM troglitazone, the ratio of albumin versus AFP expression increased consistently in a dose-dependent way (Figure 6B).

### DISCUSSION

Drugs acting through PPARγ have growth inhibitory effects on certain human cancers. Both thiazolidinedione and nonthiazolidinedione ligands have been shown to exert the inhibitory effect. In this study, we evaluated the initial aspects of growth inhibitory potential of PPARγ agonists on human HCC cell lines using the commonly studied TZD ligand troglitazone, a nonhepatotoxic TZD pioglitazone and the endogenous ligand 15d-$PGJ_2$. We found that human HCC cell lines Hep G2, HuH-7, KYN-1 and KYN-2 express PPARγ and the expressed PPARγ was functionally active. PPARγ ligands also induced growth inhibition in all the HCC cell lines. Compared to the rate of cell-count increment in drug-free control, troglitazone as well as pioglitazone and 15d-$PGJ_2$ caused a significant inhibition in cell growth. Inhibition of cell proliferation was also evident in $^3$H-thymidine incorporation assays indicating that PPARγ ligands induced inhibition of DNA synthesis in HCC cell lines. A similar dose response in inhibition of thymidine incorporation was also reported on colon cancer cells (Kitamura et al, 1999).

Regarding the dose response of troglitazone in this study, a significant growth inhibition could be obtained at 1 µM in 3 of the 4 cell lines, with marked inhibition in all at 5 µM. The other 2 PPARγ ligands also exhibited similar dose responses. According to the pharmacokinetics of troglitazone (Ploskar and Faulds, 1999) and due to a higher tissue distribution in liver (Kawai et al, 1997), a daily dose of 600 to 800 mg troglitazone may attain an effective drug level in vivo. Similarly, 30 to 60 mg daily dose of pioglitazone would result in an effective drug level. In a recent clinical trial on prostate cancer patients, 800 mg daily dose of troglitazone has already been used (Mueller et al, 2000).

Although the exact mechanism of growth inhibition of tumour cells by PPARγ ligands is not well-understood (Gelman et al, 1999), it was reported to be associated with alteration in the cell cycle. Activation of PPARγ resulted in G1 cell cycle arrest in colon cancer cells (Brockman et al, 1998; Kitamura et al, 1999), pancreatic cancer cells (Motomura et al, 2000) and leukaemia cells (Asou et al, 1999; Sugimura et al, 1999). We found that all PPARγ ligands dose dependently increased Hep G2 cells accumulating in G1 phase and decreased in S phase. Moreover, 15d-$PGJ_2$ and to a lesser extent troglitazone, induced also G2 cell cycle arrest. A G2 arrest in addition to G1 cell cycle arrest may be responsible for the higher growth inhibitory potential of 15d-$PGJ_2$ and troglitazone over pioglitazone.

To reveal the underlying mechanism of troglitazone-induced cell cycle arrest, CDK inhibitor $p27^{Kipl}$, $p21^{Cipl/Wafl}$ and $p18^{Ink4c}$ protein levels were studied in Hep G2 cells. A dose-dependent increase in $p27^{Kipl}$ and $p18^{Ink4c}$ with decreased $p21^{Cipl/Wafl}$ level was observed 48 h after troglitazone treatment. The exact mechanism of cell cycle withdrawal induced by PPARγ ligands is not yet clear but it was found related to up-regulation of $p21^{Cipl/Wafl}$ in eosinophilic leukaemia cell line EoL-1 (Sugimura et al, 1999). In a recent study, troglitazone induced G1 arrest in pancreatic carcinoma cell line

© 2001 Cancer Research Campaign 

PK-1 was found to be associated with increased p27[Kipl] but unchanged p21[Cipl/Wafl] and p18[Ink4c] levels (Motomura et al, 2000). Cell cycle withdrawal during PPARγ induced adipogenesis was also found associated with an initial increase in both p27[Kipl] and p21[Cipl/Wafl], followed by a decline in p21[Cipl/Wafl], increase in p18[Ink4c] with sustained p27[Kipl] level (Morrison and Farmer, 1999). Difference in the extent of G1 or G2 cell cycle arrest in Hep G2 cells with troglitazone, pioglitazone or 15d-$PGJ_2$ may be due to differences in cascade expression of the CDK inhibitors. However, CDK inhibitors other than p27[Kipl], p21[Cipl/Wafl] and p18[Ink4c] may also be involved in the PPARγ ligands induced cell cycle arrest. Possibility of an initial increased p21[Cipl/Wafl] followed by declined level in troglitazone treated Hep G2 cells as reported in coupling growth arrest and adipocyte differentiation (Morrison and Farmer, 1999) can not be excluded until further time course studies are done. Further studies are also required to reveal whether inhibition of the p27[Kipl] or p18[Ink4c] increase could inhibit the growth arrest in Hep G2 as found in PK-1 cells (Motomura et al, 2000).

PPARγ ligands have been shown to drive differentiation process in various malignant cells (Tontonoz et al, 1997; Kubota et al, 1998; Sarraf et al, 1998; Chang and Szabo, 2000). When we investigated the expression of AFP and albumin in Hep G2 cells after treatment with troglitazone, we found that there was a dose-dependent decrease in the AFP expression. The expression of albumin showed a little increase with 5 and 10 μM troglitazone but decrease with 25 μM. The decreased albumin expression at 25 μM may be due to associated cytotoxic response of troglitazone on Hep G2 cells. However, the inhibition of AFP expression was more prominent over albumin inhibition and results of repeated Northern analysis revealed that instead of variation in the amount of decrease in AFP expression or increase in albumin expression, the dose-dependent increase in ratio of albumin and AFP expression remained consistent.

Hepatocellular carcinoma is one of the most lethal malignancies where chemoprevention is recommended and different type newer agents are already tried on human subjects (Oka et al, 1995; Muto et al, 1996; Jacobson et al, 1997). PPARγ ligands exhibited a marked growth inhibitory potential on hepatocellular carcinoma cells through induction of G1 cell cycle arrest and recent studies have also shown that activation of PPARγ can inhibit the profibrogenic and proinflammatory actions of hepatic stellate cells (Galli et al, 2000; Marra et al, 2000; Miyahara et al, 2000). Taken together, we conclude that PPARγ ligands may also prove beneficial for primary or secondary chemoprevention of hepatocellular carcinoma. However, further studies will be necessary to evaluate their safety and therapeutic potential.

### REFERENCES

Aden DP, Fogel A, Plotkin S, Damjanov I and Knowels BB (1979) Controlled synthesis of HBsAg in a differentiated human liver carcinoma-derived cell line. *Nature* **282**: 615–616

Alam J and Cook JL (1990) Reporter genes: Application to the study of mammalian gene transcription. *Anal Biochem* **188**: 245–254

Asou H, Verbeek W, Williamson E, Elstner E, Kubota T, Kamada N and Koeffler HP (1999) Growth inhibition of myeloid leukaemia cells by troglitazone a ligand for peroxisome proliferator activated receptor gamma and retinoids. *Int J Oncol* **15**: 1027–1031

Auboeuf D, Rieusset J, Fajas L, Vallier P, Frering V, Riou JP, Stales B, Auwerx J, Laville M and Vidal H (1997) Tissue distribution and quantification of the expression of mRNAs of peroxisome proliferator-activated receptors and liver-X receptor-α in humans: No alteration in adipose tissue of obese and NIDDM patients. *Diabetes* **46**: 1319–1327

Brasier AR, Tate JE and Habener JF (1989) Optimised use of the firefly luciferase assay as a reporter gene in mammalian cell lines. *Bio Techniques* **7**: 1116–1122

Brockman JA, Gupta RA and DuBois RN (1998) Activation of PPARγ leads to inhibition of anchorage independent growth of human colorectal cancer cells. *Gastroenterology* **115**: 1049–1055

Butler R, Mitchell SH, Tindall DJ and Young CY (2000) Nonapoptotic cell death associated with S-phase arrest of prostate cancer cells via the peroxisome proliferator-activated receptor γ ligand 15-deoxy-delta 12, 14-postaglandin J2. *Cell Growth Differ* **11**: 49–61

Chang T-H and Szabo E (2000) Induction of differentiation and apoptosis by ligands of peroxisome proliferator-activated receptor γ in non-small cell lung cancer. *Cancer Res* **60**: 1129–1138

Chawla A, Schwarz EJ, Dimaculangan DD and Lazar MA (1994) Peroxisome proliferator-activated receptor (PPAR) γ: adipose-predominant expression and induction early in adipocyte differentiation. *Endocrinology* **135**: 798–800

Clay CE, Namen AM, Atsumi G, Willingham MC, High KP, Kute TE, Trimboli AJ, Fonteh AN, Dawson PA and Chilton FH (1999) Influence of J series prostaglandins on apoptosis and tumorigenesis of breast cancer cells. *Cancinogenesis* **20**: 1905–1911

Demetri GD, Fletcher CDM, Mueller E, Sarraf P, Naujoks R, Campbell N, Spiegelman BM and Singer S (1999) Induction of solid tumour differentiation by the peroxisome proliferator-activated receptor γ ligand troglitazone in patients with liposarcoma. *Proc Natl Acad Sci USA* **96**: 3951–3956

Elbrecht A, Chen Y, Cullinan CA, Hayes N, Leibowitz MD, Moller DE and Berger J (1996) Molecular cloning, expression and characterisation of human peroxisome proliferator-activated receptors γ1 and γ2. *Biochem Biophys Res Commun* **224**: 431–437

Elstner E, Muller C, Koshizuka K, Williamson EA, Park D, Asou H, Shintaku P, Said JW, Heber D and Koeffler HP (1998) Ligands for peroxisome proliferator-activated receptor γ and retenoic acid receptor inhibit growth and induce apoptosis of human breast cancer cells in vitro and in BNX mice. *Proc Natl Acad Sci USA* **95**: 8806–8811

Forman BM, Tontonoz P, Chen J, Brun RP, Spiegelman BM and Evans RM (1995) 15-deoxy-$\Delta^{12,14}$-prostaglandin J2 is a ligand for the adipocyte determination factor PPARγ. *Cell* **83**: 803–812

Galli A, Crabb D, Price D, Ceni E, Salzano R, Surrenti C and Casini A (2000) Peroxisome proliferator-activated receptor γ transcriptional regulation is involved in platelet-derived growth factor-induced proliferation of human hepatic stellate cells. *Hepatology* **31**: 101–108

Gelman L, Fruchart JC and Auwerx J (1999) An update on the mechanisms of action of the peroxisome proliferator-activated receptors (PPARs) and their roles in inflammation and cancer. *Cell Mol Life Sci* **55**: 932–943

Guan YF, Zhang YH, Breyer RM, Davis L and Breyer MD (1999) Expression of peroxisome proliferator-activated receptor γ (PPARγ) in human transitional bladder cancer and its role in inducing cell death. *Neoplasia* **1**: 330–339

He TC, Chan TA, Vogelstein B and Kinzler KW (1999) PPARγ is an APC-regulated target of nonsteroidal anti-inflammatory drugs. *Cell* **99**: 335–345

Henney JE (1999) From the Food and Drug Administration: new type 2 diabetes drugs. *JAMA* **282**: 932

Hirase N, Yanase T, Mu Y, Muta K, Umemura T, Takayanagi R and Nawata H (1999) Thiazolidinedione induces apoptosis and monocytic differentiation in the promyelocytic leukemia cell line HL60. *Oncology* **57(S2)**: s17–s26

Hisatake JI, Ikezoe T, Carey M, Holden S, Tomoyasu S and Koeffler HP (2000) Down-regulation of prostate-specific antigen expression by ligands for peroxisome proliferator-activated receptor γ in human prostate cancer. *Cancer Res* **60**: 5494–5498

Jacobson LP, Zhang BC, Zhu YR, Wang JB, Wu Y, Zhang QN, Yu LY, Qian GS, Kuang SY, Li YF, Fang X, Zarba A, Chen B, Enger C, Davidson NE, Gorman MB, Gordon GB, Prochaska HJ, Egner PA, Groopman JD, Munoz A, Helzlsouer KJ and Kensler TW (1997) Oltipratz chemoprevention trial in Qidong, People's Republic of China: study design and clinical outcomes. *Cancer Epidemiol Biomarkers Prev* **6**: 257–265

Juge-Aubry C, Pernin A, Favez T, Burger AG, Wahlis W, Meier CA and Desvergne B (1997) DNA binding properties of peroxisome proliferator-activated receptor subtypes on various natural peroxisome proliferator response elements. *J Biol Chem* **272**: 2525–25259

Kawai K, Kawasaki-Tokui Y, Odaka T, Tsuruta F, Kazui M, Iwabuchi H, Nakamura T, Kinoshita T, Ikeda T, Yoshioka T, Komai T and Nakamura K (1997) Disposition and metabolism of the new oral antidiabetic drug troglitazone in rats, mice and dogs. *ArzneimForsch/Drug Res* **47**: 356–368

Keelan JA, Sato TA, Marvin KW, Lander J, Gilmour RS and Mitchell MD (1999) 15-deoxy-delta (12, 14)-postaglandin J(2), a ligand for peroxisome proliferator-activated receptor γ, induces apoptosis in JEG3 choriocarcinoma cells. *Biochem Biophys Res Commun* **262**: 579–585

© 2001 Cancer Research Campaign

Kitamura S, Miyazaki Y, Shinomura Y, Knodo S, Kanayama S and Matsuzawa Y (1999) Peroxisome proliferator-activated receptor γ induces growth arrest and differentiation markers of human colon cancer cells. *Jpn J Cancer Res* **90**: 75–80

Kliewer SA and Willson TM (1998) The nuclear receptor PPARγ-bigger than fat. *Curr Opin Genet Dev* **8**: 576–581

Kliewer SA, Umesono K, Noonan DJ, Heyman RA and Evans RM (1992) Convergence of 9-cis retinoic acid and peroxisome proliferator signalling pathways through heterodimer formation of their receptors. *Nature* **358**: 771–774

Kliewer SA, Lenhard JM, Willson TM, Patel I, Morris DC and Lehmann JM (1995) A prostaglandin J2 metabolite binds peroxisome proliferator-activated receptor γ and promotes adipocyte differentiation. *Cell* **83**: 813–819

Kliewer SA, Sundseth SS, Jones SA, Brown PJ, Wisely GB, Koble C, Devchand P, Wahli W, Willson TM, Lenhard JM and Lehmann JM (1997) Fatty acids and eicosanoids regulate gene expression through direct interactions with peroxisome proliferator-activated receptor α and γ. *Proc Natl Acad Sci USA* **94**: 4318–4323

Kohlroser J, Mathai J, Reichheld J, Banner BF and Bonkovsky HL (2000) Hepatotoxicity due to troglitazone: Report of two cases and review of adverse events reported to the United States Food and Drug Administration. *Am J Gastroenterol* **95**: 272–276

Kubota T, Koshizuka K, Williamson EA, Asou H, Said JW, Holden S, Miyoshi I and Koeffler HP (1998) Ligand for peroxisome proliferator-activated receptor γ (troglitazone) has potent antitumor effect against human prostate cancer both *in vitro* and *in vivo*. *Cancer Res* **58**: 3344–3352

Lehmann JM, Moore LB, Smith-Oliver TA, Wilkinson WO, Willson TM and Kliewer SA (1995) An antidiabetic thiazolidinedione is a high affinity ligand for peroxisome proliferator-activated receptor gamma. *J Biol Chem* **270**: 12953–12956

Lehmann JM, Lenhard JM, Oliver BB, Ringold GM and Kliewer SA (1997) Peroxisome proliferator-activated receptors α and γ are activated by indomethacin and other nonsteroidal anti-inflammatory drugs. *J Biol Chem* **272**: 3406–3410

Maekawa T, Kinoshita Y, Matsushima Y, Okada A, Fukui H, Waki S, Kishi K, Kawanami C, Nakata H, Hassan S, Wakatsuki Y, Ota H, Amano K, Nakao M and Chiba T (1997) *Helicobacter pylori* induces proinflammatory cytokines and major histocompatibility complex class II antigen in mouse gastric epithelial cells. *J Lab Clin Med* **130**: 442–449

Mangelsdorf DJ, Thummel C, Beato M, Herrlich P, Schuetz G, Umesono K, Blumberg B, Kastner P, Mark M, Chambon P and Evans RM (1995) The nuclear receptor superfamily: the second decade. *Cell* **83**: 835–839

Marra F, Efsen E, Romanelli RG, Caligiuri A, Pastacaldi S, Batignani G, Bonacchi A, Caporale R, Laffi G, Pinzani M and Gentilini P (2000) Ligands of peroxisome proliferator-activated receptor g modulate profibrogenic and proinflammatory actions in hepatic stellate cells. *Gastroenterology* **119**: 466–478

Miyahara T, Schrum L, Rippe R, Xiong S, Yee Jr. HF, Motomura K, Anania FA, Willson TM and Tsukamoto H (2000) Peroxisome proliferator-activated receptors and hepatic stellate cell activation. *J Biol Chem* **275**: 35715–35722

Morrison RF and Farmer SR (1999) Role of PPARγ in regulating a cascade expression of cyclin-dependent kinase inhibitors, p18(INK4c) and p21(Waf1/Cip1), during adipogenesis. *J Biol Chem* **274**: 17088–17097

Motomura W, Okumura T, Takahashi N, Obara T and Khogo Y (2000) Activation of peroxisome proliferator-activated receptor γ by troglitazone inhibits cell growth through the increase of p27Kip1 in human pancreatic carcinoma cells. *Cancer Res* **60**: 5558–5564

Mueller E, Sarraf P, Tontonoz P, Evans RM, Martin KJ, Zhang M, Fletcher C, Singer S and Spiegelman BM (1998) Terminal differentiation of human breast cancer through PPAR γ. *Mol Cell* **1**: 465–470

Mueller E, Smith M, Sarraf P, Kroll T, Aiyer A, Kaufman DS, Oh W, Demetri G, Figg WD, Zhou XP, Eng C, Spiegelman BM and Kantoff PW (2000) Effects of ligand activation of peroxisome proliferator-activated receptor γ in human prostate cancer. *Proc Natl Acad Sci USA* **97**: 10990–10995

Muto Y, Morwaki H, Ninomiya M, Adachi S, Saito A, Takasaki KT, Tanaka T, Tsurumi K, Okuno M, Tomita E, Nakamura T and Kojima T (Hepatoma Prevention Study Group) (1996) Prevention of second primary tumours by an acyclic retinoid, polyprenoic acid, in patients with hepatocellular carcinoma. *N Engl J Med* **334**: 1561–1567

Nakabayashi H, Taketa T, Miyano K, Yamane T and Sato J (1982) Growth of human hepatoma cell lines with differentiated functions in chemically defined medium. *Cancer Res* **42**: 3858–3863

Niwa Y, Matsumura M, Shiratori Y, Imamura M, Kato N, Shina S, Okudaira T, Ikeda Y, Inoue T and Omata M (1996) Quantification of α-fetoprotein and albumin messenger RNAs in human hepatocellular carcinoma. *Hepatology* **23**: 1384–1392

Nordeen SK (1988) Luciferase reporter gene vectors for analysis of promoters and enhancers. *Bio Techniques* **6**: 454–457

Oka H, Yamamoto S, Kuroki T, Harihara S, Marumo T, Kim SR, Monna T, Kobayashi K and Tango T (1995) Prospective study of chemoprevention of hepatocellular carcinoma with Sho-saiko-to (TJ-9). *Cancer* **76**: 743–749

Plosker GL and Faulds D (1999) Troglitazone: a review of its use in the management of type 2 diabetes mellitus. *Drugs* **57**: 409–438

Sarraf P, Mueller E, Jones D, King FJ, DeAnglo DJ, Partridge JB, Holden SA, Chen LB, Singer S, Fletcher C and Spiegelman BM (1998) Differentiation and reversal of malignant changes in colon cancer through PPARγ. *Nature Med* **4**: 1046–1052

Sato H, Ishihara S, Kawashima K, Moriyama N, Suetsugu H, Kazumori H, Okuyama T, Rumi MA, Fukuda R, Nagasue N and Kinoshita Y (2000) Expression of peroxisome proliferator-activated receptor (PPAR) γ in gastric cancer and inhibitory effects of PPARγ agonists. *Br J Cancer* **83**: 1394–1400

Sugimura A, Kiriyama Y, Nochi H, Tsuchiya H, Tamoto K, Sakurada Y, Ui M and Tokumitsu Y (1999) Troglitazone suppress cell growth of myeloid leukemia cell lines by induction of p21WAF1/CIP1 cyclin-dependent kinase inhibitor. *Biochem Biophys Res Commun* **261**: 833–837

Takahashi N, Okumura T, Motomura W, Fujimoto Y, Kawabata I and Kohgo Y (1999) Activation of PPARγ inhibits cell growth and apoptosis in human gastric cancer cells. *FEBS Letter* **455**: 135–139

Tontonoz P, Hu E, Graves A, Budavari AI and Spiegelman BM (1994a) mPPARγ2: tissue-specific regulator of an adipocyte enhancer. *Genes Dev* **8**: 1224–1234

Tontonoz P, Hu E and Spiegelman BM (1994b) Stimulation of adipogenesis in fibroblasts by PPARγ2, a lipid-activated transcription factor. *Cell* **79**: 1147–1156

Tontonoz P, Singer S, Forman BM, Sarraf P, Fletcher JA, Fletcher C, Brun RP, Mueller E, Altiok S, Oppenheim H, Evans RM and Spiegelman BM (1997) Terminal differentiation of human liposarcoma cells by ligands for peroxisome proliferator-activated receptor γ and retinoid X receptor. *Proc Natl Acad Sci USA* **94**: 237–241

Tsubouchi Y, Sano H, Kawahito Y, Mukai S, Yamada R, Kohno M, Inoue K, Hla T and Kondo M (2000) Inhibition of human lung cancer cell growth by the for peroxisome proliferator-activated receptor γ agonists through induction of apoptosis. *Biochem Biophys Res Commun* **270**: 400–405

Vidal-Puig AJ, Considine RV, Jimenez-Linan M, Werman A, Pories WJ, Caro JF and Flier JS (1997) Peroxisome proliferator-activated receptor gene expression in human tissues. Effects of obesity, weight loss, and regulation by insulin and glucocorticoids. *J Clin Invest* **99**: 2416–2422

Watkins PB and Whitcomb RW (1998) Hepatic dysfunction associated with troglitazone. *N Engl J Med* **388**: 916–917

Xu HE, Lambert MH, Montana VG, Parks DJ, Blanchard SG, Brown PJ, Sternbach DD, Lehmann JM, Wisely GB, Willson TM, Kliewer SA and Milburn MV (1999) Molecular recognition of fatty acids by peroxisome proliferator-activated receptors. *Mol Cell* **3**: 397–403

Yano H, Kojiro M and Nakashima T (1986) A new human hepatocellular carcinoma cell line (KYN-1) with a transformation to adenocarcinoma. *In Vitro Cell Dev Biol* **22**: 637–646

Yano H, Maruiwa M, Murakami T, Fukuda K, Ito Y, Sugihara S and Kojiro M (1988) A new human pleomorphic hepatocellular carcinoma cell line, KYN-2. *Acta Pathol Jpn* **38**: 953–966

*© 2001 Cancer Research Campaign* 
